# Residual host cell proteins: sources, properties, detection methods and data acquisition modes

**DOI:** 10.3389/fmicb.2025.1658366

**Published:** 2025-08-18

**Authors:** Yifan Yao, Xuemei Wen, Hongjuan Pan, Ziwei Chen

**Affiliations:** 1 China State Institute of Pharmaceutical Industry, Shanghai, China; 2 National Key Laboratory of Lead Druggability Research, Shanghai Institute of Pharmaceutical Industry, Shanghai, China; 3 School of Food and Biological Engineering, Jiangsu University, Zhenjiang, China

**Keywords:** host cell proteins, sources, characterization, pretreatment, detection

## Abstract

Host cell proteins (HCPs) are process-related impurities derived from host organisms used for recombinant protein production in biopharmaceutical manufacturing. The generation of HCPs may lead to potential safety risks, such as immunogenicity, reduced drug efficacy and long-term side effects. Therefore, in the biopharmaceutical process, even trace amounts of HCPs need to be strictly regulated and controlled. The main bottlenecks associated with the detection of HCPs include a wide dynamic range of detection and instability of HCPs. Due to its high sensitivity and high resolution, mass spectrometry has attracted more and more attention in HCP detection, but it still cannot completely replace enzyme-linked immunosorbent assay (ELISA). The research in the future includes the development of more efficient sample pretreatment methods and data processing techniques to improve the sensitivity and accuracy of detection. At the same time, combined with risk assessment and process optimization, it is expected to further reduce the residual risk of HCP. This review discusses the sources, properties, pretreatment and detection of residual HCPs in therapeutic products, along with current regulatory considerations and future advancements.

## 1 Introduction

Host cell proteins (HCPs) are process-related impurities which are expressed by host cells during the production of biologic drug proteins (Hogwood et al., 2013; Wang X. et al., 2020). In the purification process of the target protein, most of the HCPs are removed (>99%). However, a small number of residual HCPs remain in the final product. HCP contamination leads to several risks to drug safety and efficacy (Beatson et al., 2011; Bergmann et al., 2012; Ngolong Ngea et al., 2021). Residual HCPs would cause immunogenicity in the body, such as allergic reactions. They may provoke an immune response in patients, leading to adverse reactions or reduced therapeutic effectiveness, which is particularly concerning patients receiving long-term treatments (Capito et al., 2013; Liang et al., 2018). Residual HCPs can affect the stability of the product, resulting in the shape and efficacy of the product. Certain HCPs, such as proteases, lipases, or oxidases, can degrade the therapeutic protein over time, reducing shelf-life and potency (He et al., 2019; Schenauer et al., 2012). Residual HCPs can interfere with the biological activity of the drug in the human body. This is because HCPs may interfere with drug mechanisms or assays, altering therapeutic outcomes. Some residual HCPs are even toxic, causing harm to patients. Enzymatic or other functional HCP contaminants can have unintended biological effects. Therefore, it is necessary to characterize and quantify residual HCPs in the bulk drug and intermediates in the downstream purification process.

The number of residual HCPs is an important evaluation index for process stability monitoring, and an important quality control index for recombinant vaccines and recombinant antibody drugs. The content of residual HCPs in biological products is generally considered as a key quality attribute of products (Mazzoni et al., 2019; Vanderlaan et al., 2018). Regulatory authorities, including the U.S. Food and Drug Administration and the European Medicines Agency, mandate strict limits for residual HCPs in therapeutic products. Key regulatory considerations include HCPs quantification, validation of assays, and risk assessment. In terms of HCP quantification, the limits vary according to the specific product and host system, typically ranging from less than 100 parts per million (ppm). For the validation of assays, ELISA or mass spectrometry-based methods which are used to detect, must be validated for accuracy, sensitivity, and robustness. Risk assessment is also an important aspect. Manufacturers need to evaluate and document the safety risks associated with detected HCPs. The International conference on harmonization guideline Q6B, the primary reference for biopharmaceutical product specifications, states that HCP levels should be minimized and well controlled. However, it does not provide exact limits while below 100 ppm is a common target used across the industry.

The detection of residual HCPs presents several challenges. First of all, the wide dynamic range (5–6 orders of magnitude) needed to detect HCPs at 1–10 ppm levels in the presence of the dominant therapeutic proteins (Ji et al., 2023; Tscheliessnig et al., 2013; Wang et al., 2019). To overcome this challenge, considerable efforts have been made to optimize the sample preparation to improve the dynamic range, such as online or offline fractionation, removal of monoclonal antibody (mAbs) by affinity depletion (Johnson et al., 2020; Madsen et al., 2015; Thompson et al., 2014; Zhang et al., 2019) or molecular weight cut-off (Chen et al., 2020; Xiao et al., 2019), and HCP enrichment (Forbes-Hernández et al., 2020; Gao et al., 2011; Mörtstedt et al., 2020; Wang Q. et al., 2020). Although these strategies are successfully applied to detect HCPs at single-digit ppm levels, they necessitated tailor-made method development for different products and significantly longer analysis times. The most significant challenge is that HCPs are complex components composed of multiple proteins, and not each HCP can effectively detect antibodies. In addition, compared with ordinary HCP, some high-risk proteins with high enzyme activity or immunogenicity have attracted more and more attention from regulators and the industry. For this kind of HCPs, if the process removal and residue limits are not monitored, there may be greater drug safety risk. For instance, certain HCPs facilitate the degradation of polysorbate, a class of non-ionic surfactants commonly used to formulate buffers for protein stabilization but can impair the stability of protein-based pharmaceuticals (Takagi et al., 2022; Wen et al., 2020). Firstly, the content of residual HCPs is low, requiring analytical methods with high sensitivity. Secondly, the variety and structure of residual HCPs require specific analysis methods. Finally, residual HCPs may be similar to target drugs. Thus, developing a highly effective way to distinguish between host residual proteins and target drugs is extremely urgent.

## 2 Sources and properties of residual host cell proteins (HCPs)

### 2.1 Sources of HCPs

HCPs are primarily sourced from various host cells used to produce biologic drug proteins, including bacteria (e.g., *Escherichia coli*), yeasts (e.g., *saccharomyces*), mammalian cells (e.g., *Chinese hamster ovary cells*) and others. HCPs are expressed during the production process and persist in the final product (Farrell et al., 2015; Liang et al., 2020). HCPs encompass a range of functional constitutes, including metabolic enzymes, structural proteins, transporters, and other functional proteins, and can exist in a variety of subtypes and modifications. They can originate from the host cell's endogenous proteins, such as cytoskeletal components, metabolic enzymes, and transcription factors, which may be expressed and secreted into the medium alongside the target protein during the production process. Additionally, HCPs may also include those secreted by the host cells and certain structural proteins generated through processes like apoptosis and metabolism (Kornecki et al., 2017).

HCPs may be derived from endogenous proteins of the host cell, apoptosis, metabolites, or cell lysis processes. These proteins play an important role in the normal physiological activity of cells but may be co-purified with target proteins during production (Jin et al., 2016; Stollar and Smith, 2020). Host cells secrete some proteins into the medium during normal metabolic processes, and these secreted proteins may be purified together with the target proteins to become impurities in the final product. In addition, during apoptosis or metabolism, host cells release certain proteins, which may also remain in the final product as HCP impurities. During cell lysis, proteins within the cell are released into the medium and become part of the HCPs. The classification of HCPs sources is summarized in Table 1.

**Table 1:** Source classification of HCPs.

**Sources**	**Classification**	**Example**
Bacteria Fungus	*Escherichia coli*	β-galactosidase, restriction endonuclease, DNA polymerase
	*Saccharomyces cerevisiae*	Ethanol dehydrogenase, pyruvate decarboxylase, galactosidase
	*Galactosidase*	Ethanol oxidase, Catalase
Mammalian cells	Chinese hamster ovary cells	Lactic dehydrogenase, glutamine synthetase
	Mouse myeloma cells	Lactic dehydrogenase, glutamine synthetase

### 2.2 Properties of HCPs

#### 2.2.1 Species diversity of HCPs

Structural proteins are important substances that constitute the structure of cells and organisms, such as actin and tubulin have a cellular scaffolding function (Hou et al., 2017; Lynch et al., 2020). Metabolic enzymes are one of the more common HCPs, such as tricarboxylic acid cycle related enzymes and glycolytic enzymes, involved in various biochemical reactions in living organisms to regulate metabolic processes (Huang et al., 2017a; Jung et al., 2023).

#### 2.2.2 Stability and functional activities of HCPs

HCPs may have a stable structure that is difficult to remove by traditional purification processes (Smaili et al., 2021; Zhang et al., 2018). In addition, HCPs have functional activities. For example, protease activity causes degradation of target proteins, affecting drug stability. Even trace amounts of HCP may trigger an immune response in human bodies. Enzyme catalytic activity such as glycosidase and oxidase may alter the glycosylation or oxidation state of the drug (Jawa et al., 2016).

#### 2.2.3 Physical and chemical properties of HCPs

The physical and chemical properties of HCPs are varied, depending on their source and structure (Schnider et al., 2024). HCP has a wide molecular weight range from small molecular weight proteins (e.g., 10 kDa) to large molecular weight proteins (>100 kDa). For example, the TRp-cage protein is one of the smallest known proteins, consisting of 20 amino acids. The largest HCPs may be close to the size of the largest known proteins and may even exceed 35,000 amino acids. In addition, the degree of isoelectric point, hydrophobicity and glycosylation of HCP is different, which increases the difficulty of purification (Huang et al., 2019; Yau et al., 2021). The hydrophobicity of HCPs determines their solubility and stability in different solvents. More hydrophobic proteins may be more soluble in organic solvents, while hydrophilic proteins are more stable in the aqueous phase. Importantly, part host proteins have enzymatic activity that may cause degradation or aggregation of target proteins in biologics, thereby affecting the stability and efficacy of drugs (Miller, 2024; Wen et al., 2019).

## 3 Pretreatment methods of HCPs detection

### 3.1 Enrichment of HCPs

The molecular weight of mAb proteins typically exceeds 150 kDa, significantly higher than that of HCP (Zhao et al., 2022). Therefore, ultrafiltration based on molecular weight cutoff (MWCO) is an effective method for the enrichment of HCPs.

The MWCO of the ultrafiltration membrane refers to the molecular weight of the substance when the interception rate of the membrane for a known molecular weight substance reaches 90% under specified conditions (Chung et al., 2022; Golly et al., 2019). It expressed in terms of molecular weight size, also referred to as the cutting molecular weight. The dialysis membrane, which is constructed from a spongy, cross-linked polymer (Musa et al., 2019; Ronco et al., 2017). It utilizes the MWCO as an indirect measure of its pore size to detect its separation performance. Small molecules diffuse across a semi-permeable membrane from the region of higher concentration to the region of lower concentration, continuing until the concentration on both sides of the membrane is equal. A one-step MWCO filtration technique is used to efficiently isolate antibodies from most high-volume polymers. This method significantly reduces the dynamic range between HCPs and antibody drugs and improves the detection limit of low abundance HCPs in filtered samples (Chen et al., 2020).

The application of small molecule probes in determining biological targets has experienced rapid development over the past decade (Yang et al., 2010). Its primary principle involves the close binding of small molecules to target biological macromolecules. Small molecule probes utilize fluorescence, isotopes, biotin, photoaffinity groups, and solid phase carriers attached to the small molecules to effectively label or separate the bound target biological macromolecules. The identification of target proteins or changes in abundance is achieved through gel electrophoresis and mass spectrometry (Sato et al., 2010). Small molecule probes are instrumental in both activity-based protein profiling and compound-centered chemical proteomics. Chemically synthesized probes are composed of reactive groups, linking groups, and enrichment tags for capturing target active proteins (Shi et al., 2011). To establish an activity-based proteomics, Regina Kufer et al. evaluated FP-biotin and FP-desthiobiotin using samples of human colorectal cancer cells (HCCF) derived from monoclonal antibodies produced in Chinese Hamster Ovary (CHO) cells (Li et al., 2021).

### 3.2 Isolation of HCPs

The affinity depletion of HCPs is a critical strategy in downstream processing of biopharmaceutical products, where affinity chromatography is used to remove HCPs. The detection and quantification of residual HCPs after affinity depletion are essential to ensure product safety and regulatory compliance. This method uses specific affinity ligands (such as antibodies and tags) to bind to the host protein specifically and then removes the bound HCPs from the sample by physical or chemical methods. A large number of therapeutic proteins were exhausted before LC-MS/MS analysis, largely overcoming the limitation of dynamic range and detecting many HCPs.

### 3.3 Enzymatic hydrolysis of HCPs

Natural digestion is defined as the direct addition of trypsin directly to monoclonal drugs without deformation. Since natural monoclonal antibodies resist trypsin digestion, the intact monoclonal antibodies are denatured by heating and then consumed from the digestive fluid by precipitation (Li D. et al., 2020).

**Figure 1:**
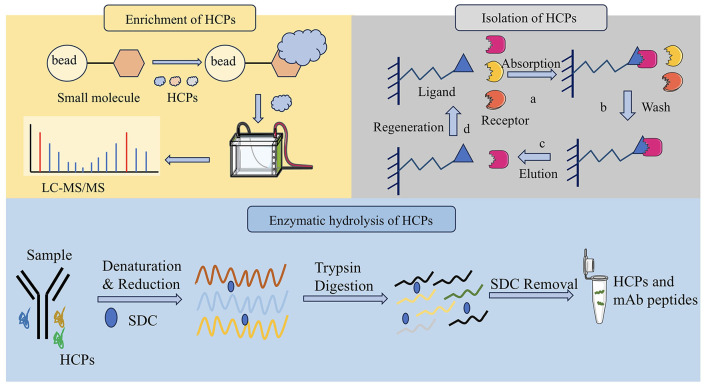
Three pretreatment methods of HCPs detection. It includes molecular weight cutoff for enrichment of HCPs, affinity depletion for isolation of HCPs and denaturing digestion for enzymatic hydrolysis of HCPs.

Denaturing digestion is the addition of denaturing agents in biological products, such as urea and guanidine hydrochloride, which can destroy the tertiary and secondary structures of proteins and denaturing them (Oyler et al., 2021). This type of digestion facilitates the enzymatic hydrolysis of proteins and subsequent analysis. Denatured digestion combined with LC-MS can provide detailed protein information, including species and content (Oyler et al., 2025). Trypsin-friendly sodium deoxycholate (SDC) is utilized as a favorable denaturant that can be effectively removed after acidification at the end of sample digestion. The two methods of denaturing digestion and natural digestion were comprehensively compared, and the results showed that the two methods complemented each other. The enrichment patterns of HCPs are summarized in Table 2 and Figure 1.

**Table 2:** Sample preparation methods for HCPs detection.

**Method type**	**Sample preparation**	**HCP enrichment mechanism**
HCPs enrichment	Molecular weight cutoff	Separation of drugs and HCPs by molecular size
	Small molecular probes	Affinity labeling covalently modifies the active site of the enzyme
HCPs isolation	Affinity deletion	Affinity chromatography
HCPs enzymatic hydrolysis	Nature digestion	Denatured reductive alkylation and enzyme digestion
	Denaturing digestion	Sodium deoxycholate

## 4 Detection methods of HCPs

### 4.1 Enzyme-linked immunosorbent assay (ELISA)

This method is a standard method for measuring total HCP concentration in multinational pharmacopeia and has the characteristics of high efficiency and convenience. ELISA plays a crucial role in the detection and quantification of residual HCPs in biopharmaceutical production processes (Palmieri et al., 2011). Its sensitivity, quantitative capability, and high throughput make it a preferred choice for routine monitoring and quality assurance. Although ELISA is simple, rapid, highly sensitive and high-throughput, it is a total content detection method, which cannot identify and analyze the type and abundance of HCPs, especially for a single high-risk protein (Van Manen-Brush et al., 2020). CHO HCP ELISA kit 3G was applied to determine the total HCP content of monoclonal antibody APIs. The HCP residues in several monoclonal antibody process intermediates and APIs were identified by LC-MS-based proteomics method with limited enzyme digestion and HCP enrichment (Huang et al., 2017b).

There are several formats used for ELISAs. These fall into either direct, indirect, or sandwich capture and detection methods. The direct ELISA method is the earliest and simplest technique. The antigen to be detected is directly coated in the micropores of the ELISA plate, and the corresponding enzyme-labeled antibody and substrate are added to make it produce color reaction (Garg et al., 2022). Indirect methods are often used to detect antibodies. The antigen is fixed to the ELISA plate, the primary antibody is added, and the antigen is specifically bound. Then, the second antibody with enzyme labels is added, so that the second antibody is specifically bound to the primary antibody. Finally, the substrate is added, making the substrate react with the enzyme to develop color, and the content of the total target protein can be determined.

Sandwich method is divided into double antibody sandwich method and double antigen sandwich method, which is suitable for the detection of macromolecular proteins with multiple recognition sites. Double antibody sandwich ELISA is often used to detect antigens. The antibody is fixed on the solid phase carrier, the antigen to be measured is added, the antibody is specifically bound to the antibody, and the enzyme-labeled antibody is added for detection, and the content of the total target protein can be determined by using the substrate color development (Sakamoto et al., 2018). The reaction mode of double antigen sandwich ELISA is similar to that of double antibody sandwich ELISA. Solid phase antigen and enzyme-specific antigen are used instead of solid phase antibody and enzyme-specific antibody respectively to determine the antibody in the sample (Li M. et al., 2020).

Competitive ELISA is suitable for the determination of small molecules with only one recognition site (Hayrapetyan et al., 2023). The principle is that the antigen in the specimen and a certain amount of enzyme-labeled antigen is competitively bound to the solid phase antibody. The more antigens in the specimen, the less enzyme-labeled antigens are bound to the solid phase, and the final color is lighter. It should be noted that the color rendering results are inversely proportional to the amount of antigen (or antibody) to be tested (Table 3).

**Table 3:** Compared direct ELISA, indirect ELISA, Sandwich ELISA with Competitive ELISA.

**Methods**	**Advantages**	**Disadvantages**
Direct ELISA	a. Fewer experimental steps b. Fast detection speed c. Don't use secondary antibodies	a. High experimental background b. Low sensitivity
Indirect ELISA	a. More immune reactivity b. Higher sensitivity.	a. Higher chance of cross-reactivity b. Longer experiment period
Sandwich ELISA	a. High sensitivity, high specificity b. Antigen without prior purification	a. The antigen must have more than two antibody binding sites b. high requirements
Competitive ELISA	High data reproducibility	Overall sensitivity and specificity were poor

### 4.2 Bradford assay

The Bradford assay is a widely used method for protein quantification based on the binding of Coomassie Brilliant Blue dye to proteins. It is a valuable tool in protein quantification due to its simplicity, speed, and sensitivity (Noble and Bailey, 2009). The common method in protein staining is Bradford, which has a sensitivity of 0.2–0.5 μg, but Bradford staining has only 10–100 times the dynamic range (Kwan and Ismail, 2019). Since the assessment of HCPs ultimately relies on the identification of detected HCPS, it is essential to use a suitable staining method that enables further characterization. The biggest disadvantage of this method is the lack of specificity, the expected presence of recombinant proteins is too abundant and masks smaller bands (Wang et al., 2009).

### 4.3 Two-dimensional polyacrylamide gel electrophoresis (2D-PAGE)

2D-PAGE is a method of separating HCP into single components by gel, which can be used to qualitatively analyze the composition of HCP (Meftahi et al., 2021; Mishra et al., 2017). The limitation of this method is that there will be cases in which the abundant protein spots block the abundant low protein spots (they are not completely separated), resulting in inaccurate analysis. What's more, sensitivity depends on different dyeing techniques. In addition, 2D-PAGE combined with western blot can also identify specific HCP, but this method is not high in throughput and time consuming. However, it is useful for detecting high-abundance HCPs and provides qualitative information and protein patterns.

### 4.4 Western blotting

Western blotting is a versatile technique that can detect proteins as low as picograms, making it a powerful tool for studying protein expression and function (Srebalus Barnes and Lim, 2007). It is widely used in research and diagnostics to identify and quantify specific proteins in a variety of samples. The western blot technique typically involves separating native or denatured proteins based on their molecular weight (size) through gel electrophoresis. It transfers these separated proteins to a protein-binding membrane and blocks non-specific proteins on the membrane with a blocking reagent and subsequent detection of the targeted protein by an antibody specific to the targeted protein (Meftahi et al., 2021).

Western blotting is a widely employed technique for confirming the identity of a protein following purification or for detecting a specific protein within a complex sample. The size of the protein band detected by Western blotting can be used to confirm the molecular weight, which can be matched to known databases or predicted values.

Samples containing the target protein are initially separated using one-dimensional or two-dimensional gel electrophoresis, followed by transferring to polyvinylidene difluoride or nitrocellulose membranes. The membrane is first blocked with Bovine Serum Albumin or other proteins, which will occupy the protein binding site on the membrane. The primary antibody produced against HCPs will incubate with the membrane and form a complex with the HCPs on the membrane. The HCP-antibody complex can be detected by directly labeling the primary antibody with an enzyme such as horseradish peroxidase or a fluorescent molecule, or indirectly by labeling a secondary antibody that specifically recognizes the primary antibody.

### 4.5 Mass spectrometry determination

Mass spectrometry (MS) is a powerful analytical technique used to measure the mass-to-charge ratio (m/z) of ions. It provides detailed information about the composition, structure, and quantity of molecules, making it an essential tool in many scientific fields, including proteomics, metabolomics, environmental analysis, and biopharmaceuticals.

MS can detect the presence of many proteins in the same sample. This method can provide a degree of quantification and compare the protein content in different samples (Srebalus Barnes and Lim, 2007). However, detection of residual HCP in highly purified recombinant protein samples requires highly sensitive instruments and skilled operators. The presence of a large number of recombinant proteins can be complicated compared to low levels of HCP. This method still cannot fully detect all existing HCPs (Chen et al., 2011).

### 4.6 Liquid chromatography tandem mass spectrometry (LC-MS/MS)

As a general detection technique, MS has certain advantages over existing HCP methods in identifying all possible HCP in biopharmaceutical samples. When combined with chromatographic separation, MS is commonly used to characterize therapeutic proteins. And it also used to identify low-abundance proteins in the 3–4 order dynamic range in various samples in the field of proteomics and biomarker discovery. Quantitative methods of mass spectrometry such as parallel detection and multiple response monitoring have been developed to determine problematic HCPs levels and assess process consistency.

Compared with traditional ELISA and 2D methods, this method has the advantages of short development time, specificity and quantification of all HCP, unbiased detection of all impurity proteins, high dynamic range (up to 6 orders of magnitude), and fast adjustment. Lisa Strasser et al. used (RP) -LC-MS/MS for peptide mapping and HCP analysis. Sook Yen E et al. developed a highly sensitive LC-MS/MS-MRM method for routine monitoring of HCPs in monoclonal antibodies produced by different processes while identifying and quantifying problematic host proteins. A multi-MRM assay can quantifies two CHO lipase proteins simultaneously. It is proved that LC-MRM analysis is the preferred HCP quantification platform, which has relatively high throughput and the limit of quantification of residual HCP in DS reaches 1 ng/mg (Strasser et al., 2021).

### 4.7 Two-dimensional liquid chromatography-high resolution mass spectrometry (2D-LC/MS)

**Figure 2:**
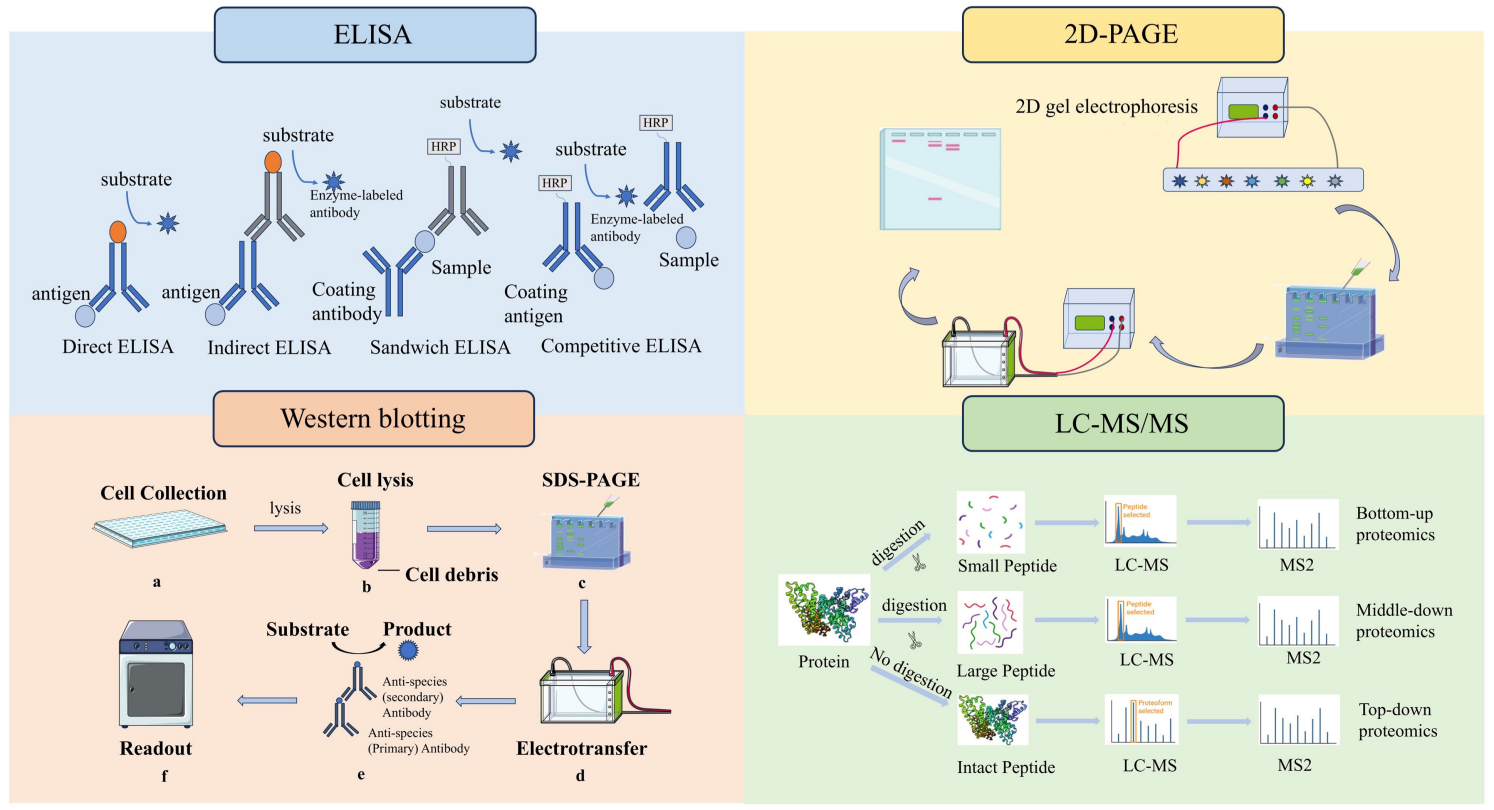
Four methods for detection of HCPs. ELISAs fall into either direct, indirect, competitive or sandwich capture and detection methods. 2D-PAGE is a method of separating HCPs into single components by gel. The western blot technique involves separating native or denatured proteins based on their molecular weight through gel electrophoresis. LC-MS is used to analyze the peptide products obtained by digesting HCPs.

Purified biologic therapeutic proteins (such as monoclonal antibodies) are digested with trypsin after reduction and alkylation and then separated by step gradient on the first dimension by reversed phase chromatography at high pH (pH 10) and then at high resolution at low pH (pH 2.5) (Yang et al., 2018). When the polypeptide is eluted from the second element, the polypeptide and its fragments are simultaneously detected by alternating between low and high energy collision cells using a quadrupole time-of-flight mass spectrometer (Doneanu et al., 2012). Doneanu et al. introduced HCP identification using comprehensive online 2D-LC/MS, followed by high-throughput HCP quantification by liquid chromatography, multiple reaction monitoring. Yang et al. present a concatenated 2D LC-MS/MS workflow that enhances reliable low-level HCP identification (92–100% success rate) at or above 10 ppm using a set of of-the-shelf protein standards as HCP surrogates spiked into a mAb drug product (Huang et al., 2021). Comparison of Detection Methods is summarized in Table 4 and Figure 2.

**Table 4:** Comparison of HCPs detection methods.

**Method**	**Sensitivity**	**Specificity**	**Quantitative**	**Advantages**	**Challenges**
ELISA	High (ng/mL)	Moderate	Yes	High throughput, quantitative	Limited antibody coverage
LC-MS/MS	Very High (pg/mL)	High	Yes	Identifies individual HCPs	Expensive, time-consuming
2D-PAGE	Moderate	Moderate	No	Visual protein profiling	Limited sensitivity
Western Blot	Moderate	High (specific HCPs)	No	Specific protein confirmation	Semi-quantitative

## 5 Data acquisition modes

Data Dependent Acquisition (DDA) and Data Independent Acquisition (DIA) are two commonly used data acquisition modes in mass spectrometry.

DDA data relies on acquisition mode, which is the most primitive and simplest data acquisition mode (Bichmann et al., 2024). After primary mass spectrometry, parent ions can be screened according to the set screening conditions for secondary mass spectrometry to obtain more fragmented information. This method has a narrow window for selecting target ions, which can reduce the presence of interfering ions to a certain extent. But it will also cause some ions with high ion peak intensity to be used as target ions for secondary mass spectrometry analysis, resulting in inaccurate sampling and poor analysis repeatability (Luque-Córdoba et al., 2024). Therefore, it is not suitable for the analysis of complex samples.

DIA data independent acquisition mode is an extension and development of DDA mode. DIA mainly collects primary mass spectrum and fragment information alternately through high and low impact energy and does not need to screen parent ions (Tiwary et al., 2019). It uses several Windows to scan the mass spectrum in full range and quickly and cyclically select and detect all ions in each window. Theoretically, it can obtain all the fragment information of all ions in the sample, improve the data utilization, less missing values, higher analytical repeatability, and more suitable for the analysis and detection of large and complex samples (Tiwary et al., 2019). With DIA it is possible to cover a higher dynamic range, and since fragment spectra for all eluting peptides are acquired irrespective of their abundance, it allows for a more comprehensive analysis without depending on prior fractionation (Molden et al., 2021).

**Figure 3:**
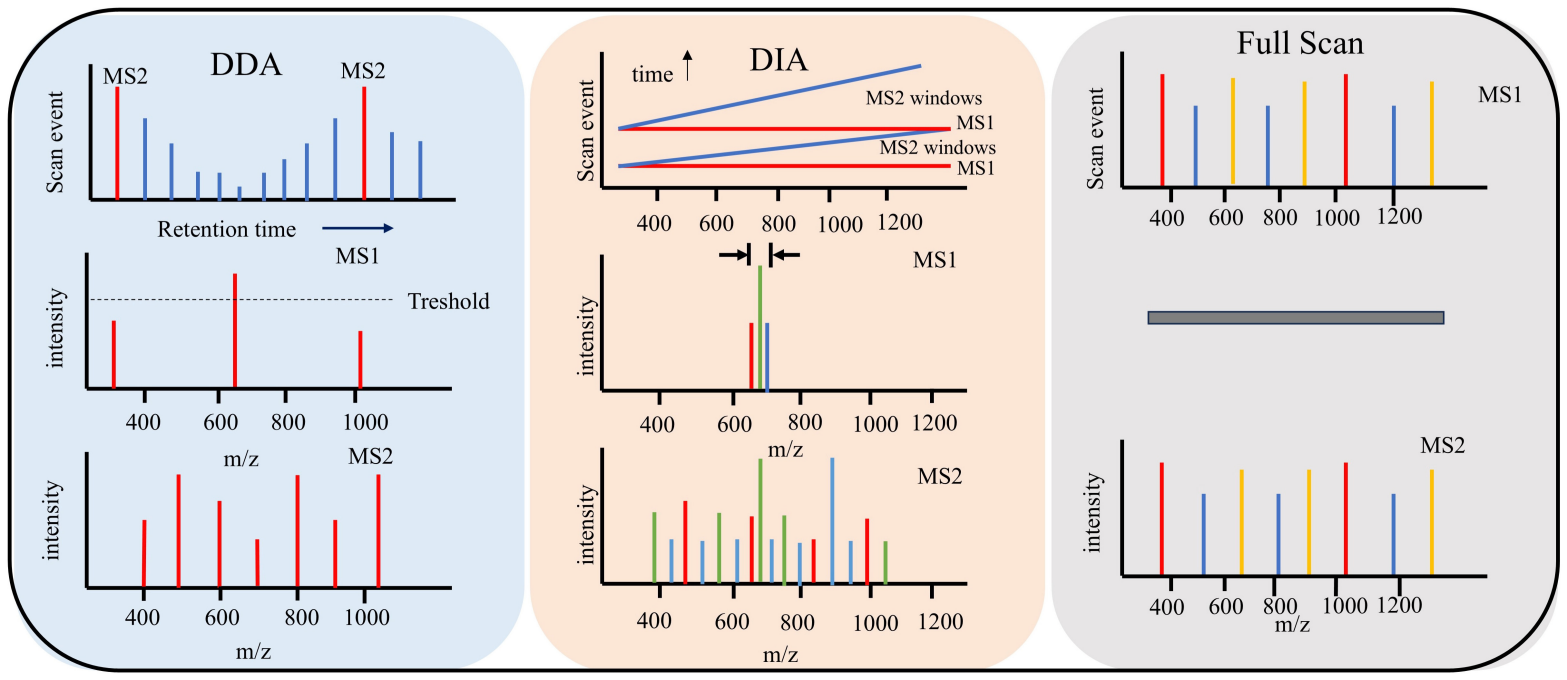
Three methods for data acquisition modes. DDA data relies on acquisition mode. DIA mainly collects primary mass spectrum and fragment information. Full scan obtains the overall mass spectrometry spectra of all ions in the sample.

The performance of a low-nanoflow UHPLC separation setup was optimized and evaluated and several LC-MS methods in data-dependent acquisition, wide-window-acquisition and wide-window data-independent acquisition to achieve deep proteome profiling (Zheng et al., 2023). A workflow was established for HCP detection and quantitation using an automated magnetic bead-based sample preparation, in combination with a DIA LC-MS analysis. Quantitation of HCPs over a broad dynamic range can monitor problematic HCPs or track changes upon altered bioprocessing conditions (Figure 3).

## 6 Conclusions

HCPs are inevitable by-products in the biopharmaceutical production process and are endogenous proteins derived from the expression system itself. With the rapid development of the biopharmaceutical industry, the detection, control and clearance of HCPs have become key links to ensure the safety and efficacy of drugs. This article elaborates in detail on the sources, properties, pretreatment methods and detection methods of residual HCPs and briefly describes the hazards of HCPs and the necessity of quality control for HCPs. Facing challenges such as the difficulty in eliminating HCPs and the need for highly sensitive detection methods for low-abundance HCPs, the current solutions mainly include cell engineering modification, multi-step chromatography, and full-spectrum analysis with high-resolution mass spectrometry. In the future, the implementation of personalized HCP control strategies will involve customizing host cells and purification processes based on drug types, such as antibodies and vaccines. Real-time monitoring of the dynamic changes of HCPs to improve batch consistency.
